# BARRIERS TO VACCINE UPTAKE IDENTIFIED BY COMMUNITY-BASED ORGANIZATIONS FOR INSITUTIONALLY UNDERSERVED GROUPS

**DOI:** 10.1093/geroni/igac059.1837

**Published:** 2022-12-20

**Authors:** Tracy Wharton, Emily Costello, Vincent Lafronza, Oscar Espinosa

**Affiliations:** National Network of Public Health Institutes, Kensington, Maryland, United States; National Network of Public Health Institutes, Atlanta, Georgia, United States; National Network of Public Health Institutes, Washington, District of Columbia, United States; National Network of Public Health Institutes, Washington, District of Columbia, United States

## Abstract

Through a competitive proposal process for a recent funding opportunity, 38 community based organizations submitted proposals that addressed vaccine hesitancy among adults who are members of racial or ethnic minority communities. Proposals were required to include discussion of barriers identified in the community of focus and evidence to support all assertions. Submissions ranged from single employee projects to large collaborative networks, and were submitted from all regions of the US, including both tribal and territorial areas, including more than 45 ethnic and language groups. Barriers were coded by two reviewers and six (6) primary themes were identified: access related to transportation, distance, or time; lack of culturally responsive materials or sensitivity of providers; structural issues such as poor data collection, historical inequity, mistrust, or systemic racism; messages coming from untrusted sources; misinformation or no information available; and differing cultural perspectives. The most commonly identified issues were related to mistrust, historical structural issues or fear and racism (n=23), and lack of access due to transportation, distance, or time (n=17). This group of proposals for funding represent a small cross-section of communities who continue to have significant pockets of unvaccinated persons. While it is possible to see themes for barriers that are encountered in increasing vaccination rates among adults, communities demonstrate extremely nuanced realities, filtered through a range of culturally and paradigmatically different ways of knowing. Success in public health initiatives requires intensive focus on the variance across these different perspectives and careful attention to appropriately focus outreach and messaging.

